# Effects of moving emergency trauma laparotomies from the ED to a dedicated OR

**DOI:** 10.1186/1757-7241-21-72

**Published:** 2013-09-23

**Authors:** Sigrid Groven, Paal Aksel Naess, Nils Oddvar Skaga, Christine Gaarder

**Affiliations:** 1 Department of Traumatology, Oslo University Hospital- Ulleval, Oslo, Norway; 2 Department of Surgery, Vestre Viken HF Drammen Hospital, Drammen, Norway; 3 Department of Anaesthesiology, Oslo University Hospital- Ulleval, Oslo, Norway; 4 Department of Traumatology, Division of Emergency and Critical Care, Oslo University Hospital- Ulleval, PO Box 4956, Nydalen N-0424, Oslo, Norway

**Keywords:** Trauma, Abdominal injury, Laparotomy, Emergency department, Survival

## Abstract

**Introduction:**

The trauma room at Oslo University Hospital- Ulleval is fully equipped for major damage control procedures, in order to minimize delay to surgery. Since 2006, patients in need of immediate laparotomy have increasingly been transferred to a dedicated trauma operating room (OR). We wanted to determine the decrease in number of procedures performed in the emergency department (ED), the effect on time from admission to laparotomy, the effect on non-therapeutic laparotomies, and finally to determine whether such a change could be undertaken without an increase in mortality.

**Methods:**

Retrospective evaluation of haemodynamically unstable trauma patients undergoing laparotomy during the period 2002–2009. Based on time for protocol change Period 1 was defined as 2002–2006 and Period 2 as 2007–2009. Significance was set at p < 0.05.

**Results:**

A total of 167 consecutive patients were included; 103 patients from Period 1 and 64 from Period 2. We found a 42% decrease in ED laparotomies (p < 0.001). Median time to laparotomy increased from 24.0 to 34.0 minutes from Period 1 to Period 2 (p = 0.029). Crude mortality fell from 57% to 39%. The proportion of non-therapeutic laparotomies in the OR tended to be lower over the whole study period.

**Conclusion:**

Moving this cohort of haemodynamically compromised trauma patients in need of emergency laparotomy out of the ED to a dedicated OR resulted in longer median time to laparotomy, but did not increase mortality.

## Introduction

Haemodynamically compromised trauma patients with suspected abdominal bleeding need immediate attention from a multidisciplinary team, and the primary aim should be to control haemorrhage. Any delay before surgery may adversely affect outcome [1,2].

Time from emergency department (ED) arrival to laparotomy has been used extensively as an audit filter in performance improvement processes [3-5]. Clarke et al. demonstrated that time to laparotomy for intra-abdominal bleeding does affect survival, increasing the risk of death by 1% for every 3 minutes delay [6].

In order to minimize delay to surgery, the treatment protocol at Oslo University Hospital-Ulleval (OUH-U) until 2006 encouraged major damage control procedures including laparotomies to be performed in a 3-bed trauma room in the ED. However, although fully equipped for major surgical procedures, conditions for operating in the ED setting are suboptimal, since the room does not meet the requirements of a formal operating room. Moreover, blocking the trauma room affects preparedness in an increasingly busy trauma center environment.

In spite of the risk of increasing time to surgery, a change in protocol was made in 2006, mandating patients in need of trauma laparotomy to be transferred to the dedicated trauma operating room (OR) one floor above the ED, when deemed possible.

The aim of this study was to detect the effect of moving emergency trauma laparotomies in patients with cardiac activity on presentation, from the trauma room in the ED to the dedicated OR. We wanted to determine the decrease in the number of laparotomies performed in the ED, the effect on time from admission to laparotomy, as well as on the non-therapeutic laparotomy rate. Finally, we wanted to determine whether this change – potentially challenging time as an accepted performance indicator – could be undertaken without an increase in mortality.

## Patients and methods

OUH-U is a major Scandinavian trauma center currently admitting approximately 1,600 trauma patients per year. Blunt trauma is the mechanism of injury in 90% of the patients. Consistently, approximately 40% are severely injured with an injury severity score (ISS) >15 [7].

The current study is a retrospective analysis of all haemodynamically unstable trauma patients undergoing laparotomy and entered into the institutional trauma registry during the period January 1, 2002 to December 31, 2009. Patients were excluded if they had undergone laparotomy at the local hospital before transfer to OUH-U. During the same period, no patient could be identified to have died of intraabdominal haemorrhage without having undergone laparotomy.

The treatment protocol throughout the study period was focused on physiology, and with intra-abdominal haemorrhage identified with DPL or FAST in haemodynamically unstable patients. Emergency room thoracotomy (ERT), with cross-clamping of the aorta followed by laparotomy when indicated is performed in the unresponsive exsanguinated patient who has shown signs of life within the last 15 minutes, and obviously severe abdominal bleeding with a systolic blood pressure (sBP) < 60 mmHg with no response to fluid resuscitation. Other patients with a transient response with suspected intra-abdominal bleeding would undergo emergency laparotomy. The patients who are haemodynamically normalizing upon initial resuscitation and with suspected abdominal injury would routinely undergo CT scan before further procedures.

The change in 2006 consisted in rapid decision-making and transfer to a dedicated OR one floor above the ED when physiology allowed. Indications for ERT remained unchanged throughout the study period [8], whereas in Period 2 further operative procedures such as laparotomy required confirmed cardiac activity, since systematic trauma auditing had revealed that some laparotomies had been performed in patients where emergency room thoracotomy with cross-clamping of the aorta had not been successful in reestablishing cardiac activity. The institutional massive transfusion protocol was updated to a balanced use of red cells, plasma, and platelets in 2007.

The patient was defined as unstable by the trauma team leader when sBP on admission was <90 mmHg and the patient was not responding adequately to initial resuscitation.

The protocol change required a change in attitude in the trauma teams and thus the change happened gradually in 2006. We therefore chose to use January 2007 as the cut-off point in time for the current study. Period 1 was thus defined as January 1, 2002 to December 31, 2006 and Period 2 as January 1, 2007 to December 31, 2009.

Data extracted from the OUH Trauma Registry included age, gender, mechanism of injury, ISS, Glasgow coma scale (GCS) score, surgical procedure codes, 30-day survival, and main cause of death based on patient charts and autopsy reports when available. Multiple organ failure (MOF) was defined as failure of two or more organ systems according to accepted definitions. The trauma registry includes all trauma patients admitted through trauma team activation (irrespective of ISS), or with penetrating injuries proximal to elbow or knee, or with ISS ≥ 9 admitted to OUH-U directly or via a local hospital within 24 hours after injury. Transfers more than 24 hours after injury are included only if the trauma team is activated. Anatomic injury was classified according to the Abbreviated Injury Scale 1998 (AIS-98) [9]. Data on surgical procedures were used to identify patients who had undergone laparotomy. Details about the surgical procedures were then extracted from the patient charts.

Laparotomies were deemed futile and were excluded if performed after emergency thoracotomy in the ED in a patient with cardiac arrest or pulseless electrical activity (PEA), and who was declared dead without having regained adequate cardiac function. Non-therapeutic laparotomy was defined as absence of intra-abdominal injury or injury not requiring intervention.

In order to assess whether the patients surviving ED laparotomy could have been transferred to the OR and whether the patients dying in the OR could have been saved with an ED laparotomy, an audit process was performed where all patient charts were reviewed by three of the authors (SG, PAN, CG). In case of different conclusions, consensus was reached by discussion.

### Statistical methods

Chi square and Fisher’s exact tests were used for analyses of categorical data, and Student’s t test and Mann–Whitney U test were used for normally (presented as mean (SD)) and non-normally (presented as median (interquartile range)) distributed continuous data, respectively. Statistical analyses were performed using PASW Statistics 18 statistical software (SPSS Inc., Chicago, USA). A *p* value of < 0.05 was considered to indicate significance.

Risk adjustment in this study was based on TRISS-methodology [10]. We employed TRISS regression coefficients published by the US National Trauma Data Bank in 2005. W-statistics, expressing excess survivors per 100 patients treated at OUH-U compared to TRISS model predictions [11] was calculated according to convention and used to compare outcomes for the two periods. Non-overlapping 95% confidence intervals were deemed as significant differences between groups.

The study was approved by the Data Protection Officer, and the Regional Committee for Medical Research Ethics had no objections.

## Results

### Period 1 vs. period 2

**Figure 1:**
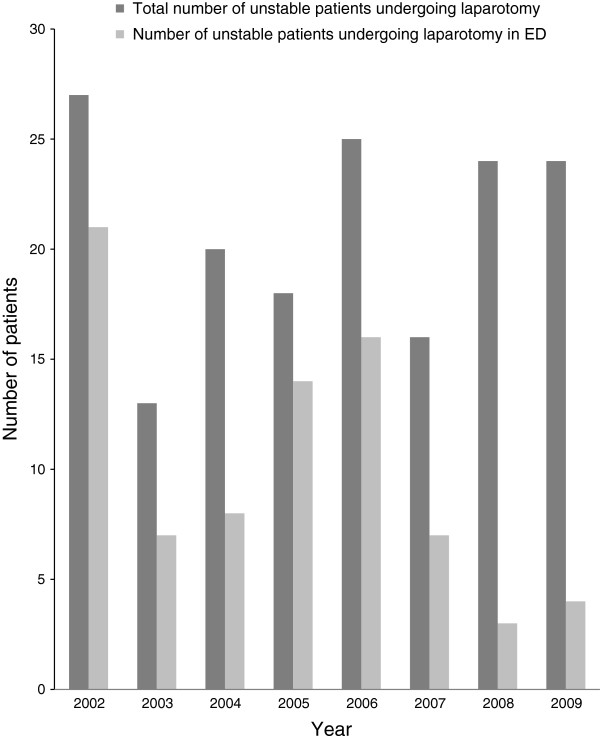
Annual number of haemodynamically unstable trauma patients undergoing laparotomy and subgroup performed in the ED.

A total of 192 unstable patients underwent laparotomy during the whole study period. Of these, 25 laparotomies were futile and the patients were thus excluded. The remaining 167 patients constitute our study population, with 103 included in Period 1 and 64 in Period 2. Patient data for the two periods were compared for demographics and outcome (Table 1). Figure 1 shows year by year numbers of unstable patients undergoing laparotomy and unstable patients undergoing ED laparotomy. Emergency room thoracotomy (ERT) was performed in 40 of the patients during Period 1 and 9 patients in Period 2.

**Table 1:** Demographics and outcome for unstable patients undergoing laparotomy in period 1 and 2

	**Period 1**	**Period 2**	
	**n = 103**	**n = 64**	***p***
Age			
mean (SD)	39.4 (20.0)	37.9 (16.7)	0.612
Male (%)	74 (72)	48 (75)	0.722
Blunt (%)	88 (85)	52 (81)	0.520
GCS score			
median (interquartile range)	6.0 (10.0)	12.0 (8.0)	0.003
Admission BP			
mean (SD)	80 (34)	76 (31)	0.473
Admission BE			
mean (SD)	−11 (7)	−11 (7)	0.609
ISS			
mean (SD)	43 (16)	36 (17)	0.011
Deaths (%)	59 (57)	25 (39)	0.026
W NTDB 05 (95% CI)	1.97 (−4.37 to 8.30)	0.22 (−8.42 to 8.86)	
Laparotomy in ED (%)	66 (64)	14 (22)	<0.001
Non-therapeutic laparotomies overall (%)	28 (27)	13 (20)	0.359
In ED (%)	19 (18)	6 (9)	0.124
In OR (%)	9 (9)	7 (11)	0.788
Time to emergency laparotomy (minutes)			
median (interquartile range)	24.0 (32.0)	34.0 (27.8)	0.029

GCS: Glascow Coma Scale; BP: systolic blood pressure; BE: base excess; ISS: injury severity score; W NTDB 05: W-statistics with coefficients from National Trauma Data Bank 2005; CI: confidence interval; ED: emergency department; OR: operating room.

Admisson BP is missing for 13 patients in Period 1 and 1 patient in Period 2. Admission BE is missing for 2 patients in Period 1 and 7 patients in Period 2.

There was a significant decrease in ED laparotomies from 64% in period 1 to 22% in period 2 (Table 1) (p < 0.001). We found a decrease in crude mortality from 57% in Period 1 to 39% in Period 2 (p = 0.026). However, when adjusting the numbers for case mix according to TRISS (W-statistics), this apparent reduction did not reach significance, as demonstrated by overlapping 95% confidence intervals (Table 1). Median time to laparotomy was 24.0 minutes in Period 1 and 34.0 minutes in Period 2 (p = 0.029) (Table 1). The appearant difference in non-therapeutic laparotomy rate between ED and OR seems to have disappeared in Period 2 (Table 1).

The rate and distribution of main causes of death remained unchanged (Table 2).

**Table 2:** Main causes of death

	**Period 1**		**Period 2**		
	**N**	**%**	**N**	**%**	p*
Bleeding	40	68	15	60	0,62
Sepsis/MOF	5	8.5	3	12	0,69
Head injury	9	15	4	16	1,00
Other/unknown	5	8.5	3	12	0,69
Total	59	100	25	100	

MOF: multiple organ failure; *Fisher’s Exact tests for individual causes against the sum of all other causes.

### ED laparotomy vs. OR laparotomy

A total of 80 patients underwent laparotomy in the ED during the study period, while 87 underwent laparotomy in the OR. Demographics are listed in Table 3. Not surprisingly, the patients undergoing laparotomy in the ED were more physiologically compromised and more severely injured. The crude mortality rate for ED laparotomy was 68% compared to 34% for patients undergoing laparotomy in the OR (p < 0.001). However, when adjusting the numbers for case mix according to TRISS (W-statistics), this apparent reduction did not reach significance (Table 3). There was a trend towards more non-therapeutic laparotomies in the ED than when performed in the OR (31% vs 18%, respectively; p = 0.072).

**Table 3:** Comparison of patients undergoing OR and ED laparotomies for the whole study period

	**Laparotomy in OR**	**Laparotomy in ED**	
	**n = 87**	**n = 80**	***p***
Age			
mean (SD)	40.0 (18.9)	37.7 (18.6)	0.434
Male (%)	63 (72)	59 (74)	0.863
Blunt (%)	71 (82)	69 (86)	0.529
GCS score			
median (interquartile range)	12.0 (8.0)	3.5 (8.0)	<0.001
Admission BP			
mean (SD)	85 (27)	70 (37)	0.003
Admission BE			
mean (SD)	−9 (5)	−13 (7)	<0.001
ISS			
mean (SD)	38 (17)	42 (16)	0.097
Deaths (%)	30 (35)	54 (68)	<0.001
W NTDB 05 (95% CI)	4.55 (−2.95 to 12.05)	−2.19 (−9.11 to 4.72)	
Non-therapeutic laparotomies, n patients (%)	16 (18)	25 (31)	0.072
Time to emergency laparotomy (minutes)			
median (interquartile range)	40.0 (29.5)	17.0 (15.3)	<0.001

ED: emergency department; OR: operating room.

GCS: Glascow Coma Scale; BP: systolic blood pressure; BE: base excess; ISS: injury severity score; W NTDB 05: W-statistics with coefficients from National Trauma Data Bank 2005; CI: confidence interval.

The audit process did not identify any of the 30 patients who died in the OR in whom a laparotomy performed in the ED would have changed outcome. Median time to OR laparotomy was 40.0 minutes. The 26 patients surviving ED laparotomy could have been safely transferred to the OR.

## Discussion

A significant decrease in emergency laparotomies performed in the ED was obtained, especially towards the end of the study period. The change could be done without a concomitant increase in mortality, in spite of an increase in median time to laparotomy. It is well known that control of bleeding is important, and therefore time to emergency laparotomy has been used as a quality indicator to assess efficiency and performance of the institution [3,12,13]. However, recent publications fail to find enough evidence to support this and to prove the validity of other commonly used quality indicators in trauma care [14-16]. Recent publications advocate operating capabilities and hybrid solutions closer to the ED due to the time aspect. A formal OR or hybrid suite in or close to the ED was not a realistic option in our institution in 2006, and was no argument against optimizing perioperative conditions when possible by moving the patient to a formal OR.

According to the review process, it is unlikely that a laparotomy performed in the ED would have changed outcome in any of the patients who died in the OR. Similarly, all 26 patients surviving ED laparotomy could have been transferred to the OR without consequences for outcome. However, given that the teams and surgical approach are the same in the ED and the OR, in some patients where ED thoracotomy had been performed, followed by therapeutic laparotomy including extraperitoneal pelvic packing, completing the operative treatment in the ED seemed practical, and justifies maintaining the capability to do so.

The trauma team’s primary task is to save lives, while hasty, futile and non-therapeutic procedures should be avoided. The surgical trauma team leader’s experience with critically injured patients will influence decision-making and outcome [17-19]. Several authors have addressed the importance of trauma surgical consultant presence in the early phase [19-21]. We have previously described the surgical trauma team leader role in our institution as filled by experienced general surgeons at the end of their surgical subspecialization, but most often with limited trauma experience [22]. The trauma team leaders attend an extensive training program, but typically stay in the role as trauma team leader for only 1.5 years due to the time limits of their training appointment. Our group also published the volume of trauma laparotomies per trauma team leader to be limited to an average of 10 per year [22]. The current findings support the need for a dedicated trauma surgical consultant presence in the early phase of the critically injured patients, as this limited experience undoubtedly also has impact on other aspects of trauma care.

This study has weaknesses in addition to the ones associated with its retrospective nature. The study addresses two consecutive periods, with the possibility of other factors influencing outcome measures as part of the ongoing quality improvement program, such as the implementation of the updated massive transfusion protocol in 2007. Another example is that on-going efforts to optimize teamwork in the ED might have counteracted the trend towards an increase in time to laparotomy from Period 1 to Period 2, as well as the decrease in laparotomies following ERT. Given that the indications for ERT remained unchanged during the study period [8], the reduction in the number of ERTs preceding laparotomy from Period 1 to Period 2 likely reflects the fact that laparotomy was no longer performed unless cardiac activity had been regained. The fact that the difference in non-therapeutic laparotomies between ED and OR seemed to have disappeared in Period 2 might have been due to better decision-making with improved educational programs. However, the numbers are too small for any firm conclusions.

Our group has recently shown that the beginning of long-lasting improvement in performance with increased survival in our total trauma population coincided with the formalization of a Trauma Service in 2005 [23]. A range of changes were made over the study period, both before and after 2005, influencing patient outcomes in general. Although not being able to adjust for all confounders, the value of critical evaluation of implemented changes should not be underestimated.

Comparison of crude mortality rates without adjusting for the risk profile of the patients is of limited value. The intention with risk adjustment is to remove sources of variation that are institution independent. Anatomic injury, physiological derangement, age, and injury mechanism are well-founded predictors of trauma outcome, all implemented in the TRISS-methodology. Thus, differences in case mix in our study are adjusted for in the survival prediction models, showing no significant difference in mortality rates. One could speculate that this difference in case-mix would explain the difference in ED laparotomy rate. However, given the comparable haemodynamics (sBP, BE), it is unlikely that the difference in GCS and ISS would account for the higher ED laparotomy rate in Period 1.

The categorization of the patients as unstable and the laparotomies as non-therapeutic was based on subjective evaluation of patient charts, but strengthened by creating consensus between three of the authors. The same applies for the evaluation of OR deaths and ED survivors. The influence of this subjectivity would likely affect both periods similarly.

## Conclusion

Moving this cohort of haemodynamically compromised trauma patients in need of emergency laparotomy out of the ED to a dedicated OR resulted in longer median time to laparotomy, but did not increase mortality.

## Competing interests

None of the authors had any conflict of interest and there were no sources of funding.

## Authors’ contributions

SG, PAN, NOS and CG designed the study. SG and NOS performed the data collection. The statistical analysis of the data was performed by SG. SG drafted the manuscript assisted by PAN, NOS and CG. All authors contributed to the interpretation of the data and writing of the manuscript. All authors revised the manuscript and approved it in the final form.
